# Assessment of the measurement properties of quality of life questionnaires in Brazilian women with breast cancer

**DOI:** 10.1590/bjpt-rbf.2014.0045

**Published:** 2014

**Authors:** Indiara S. Oliveira, Lucíola C. M. Costa, Ana C. T. Manzoni, Cristina M. N. Cabral

**Affiliations:** 1 Programa de Mestrado e Doutorado em Fisioterapia, Universidade Cidade de São Paulo (UNICID), São Paulo, SP, Brazil; 2 Curso de Fisioterapia, Universidade Cidade de São Paulo (UNICID), São Paulo, SP, Brazil

**Keywords:** breast cancer, questionnaires, quality of life, reliability and validity, physical therapy

## Abstract

**BACKGROUND:**

There are several questionnaires available to assess quality of life in breast cancer, however the choice of the best questionnaire often does not take into account the adequacy of these questionnaires' measurement properties.

**OBJECTIVE:**

To test the measurement properties of two generic quality of life questionnaires and one quality of life questionnaire specific for women with breast cancer.

**METHOD:**

We assessed 106 women after surgery for breast cancer. The assessment included application of the SF-36, WHOQOL-bref, and FACT-B+4 questionnaires as well as the Global Perceived Effect and Pain Numerical Rating scales. The participants were interviewed on three occasions to investigate internal consistency, floor and ceiling effects, construct validity, reproducibility, and responsiveness.

**RESULTS:**

Most of the instruments' domains showed adequate internal consistency (Cronbach's alpha varying from 0.66 to 0.91). Reliability varied from poor to substantial (ICC_2,1_ between 0.39 and 0.87) and agreement varied from negative to very good. The SF-36 presented doubtful agreement and showed floor and ceiling effects in three domains. The domains of the generic questionnaires presented moderate to good correlation with the FACT-B+4 (Pearson varying from 0.31 to 0.69). The internal responsiveness varied from small to large (ES varying from -0.26 to 0.98) and external responsiveness was found in only some of the instruments' domains.

**CONCLUSIONS:**

Most of the measurement properties tested for the WHOQOL-bref and FACT-B+4 were adequate as was their ability to assess quality of life in women with breast cancer. The SF-36 showed inadequacy in agreement and floor and ceiling effects and should not be used in women with breast cancer.

## Introduction

Breast cancer is a significant public health issue in Brazil, and it is considered the second most common cause of death among women^1^. After surgical treatment, the patient experiences severe physical and motor consequences that negatively influence the clinical condition. Some examples of these changes are limitation of the upper limb movements, pain and functional impairment, paresthesia, postural asymmetries, fibrosis of the glenohumeral joint, and lymphedema^2^^-^ 5. Some studies show the correlation between the treatment of breast cancer and functional impairment and demonstrate that the measurement of quality of life related to health becomes important to understand how the functional impairment interferes, in general, in the daily activities of the women diagnosed with breast cancer^6^^-^ 9.

Quality of life (QoL) assessment consists basically of questionnaires, most of which have been created in English and are aimed toward English-speaking populations^10^^-^ 12. The number of instruments available to assess QoL in cancer patients has increased and today there are several breast cancer-specific questionnaires in the literature^12^^,^ 13. The Functional Assessment of Cancer Therapy - Breast plus Arm Morbidity (FACT-B+4) is a QoL questionnaire specific for women with breast cancer. The FACT-B+4 has been already tested in the Brazilian population and showed appropriate internal consistency, reproducibility, and construct validity^14^ compared with other specific QoL questionnaires.

Additionally, generic questionnaires can be proposed for this assessment. The Medical Outcomes Study 36 - Item Short-Form Health Survey (SF-36) and World Health Organization Quality of Life - bref (WHOQOL-bref) questionnaires have been used to assess general QoL in Latin America^15^^-^ 20. However, measurement properties are not always tested in most instruments, taking into account the language and target population. To date, no published studies have completely tested the measurement properties of QoL assessment questionnaires in Brazilian-Portuguese and applied them to women with breast cancer.

Considering the choice of the most appropriate questionnaire for women with breast cancer, the aim of the present study is to test the measurement properties of the SF-36 and WHOQOL-bref compared to the FACT-B+4. The secondary objectives are to determine the preference and acceptance of the QoL questionnaire and assess its ease of comprehension. The hypothesis of this study is that the generic questionnaires available for general clinical purposes will be acceptable and will have good clinimetric results for the population of women with breast cancer when compared to the FACT-B+4.

## Method

### Sample

The study included 106 women who underwent breast cancer surgery, constituting a convenience sample that was assessed between 27 March and 28 November 2012. The inclusion criteria were: women aged 18 years or more with a primary diagnosis of breast cancer at any stage of the disease, submitted to breast cancer surgery in the last 5 years, discharged from hospital (to avoid immediate postoperative adaptations and consequent influence on the QoL), having received or currently receiving treatment with radiotherapy, chemotherapy, and/or hormone therapy, and recruited at Hospital do Câncer AC Camargo - Fundação Antônio Prudente, in the city of São Paulo, SP, Brazil. The exclusion criteria were: breast cancer as a secondary diagnosis and inability to read, write or speak fluently in Portuguese.

The participants who agreed to participate signed an informed consent form prior to data collection. The study was approved by the Research Ethics Committee of Universidade Cidade de São Paulo (UNICID), São Paulo, SP, Brazil (protocol 13616825) and by the Human Research Ethics Committee of Fundação Antônio Prudente - Hospital do Câncer AC Camargo, São Paulo, SP, Brazil (protocol 1627/11).

### Assessment instruments

### Assessment sheet

An assessment sheet was used to gather sociodemographic, clinical data, and clinical characteristics of the cancer. Some data were obtained directly from the patient's electronic medical records.

### Medical Outcomes Study 36 - Item Short - Form Health Survey (SF-36)

The SF-36^21^, adapted to Brazilian-Portuguese^22^, is a generic QoL questionnaire composed of 11 questions with 36 items divided into eight dimensions: physical functioning (questions 3 to 12), role limitations due to physical health (role-physical - questions 13 to 16), role limitations due to emotional problems (role-emotional - questions 17 to 19), bodily pain (questions 21 and 22), general health perceptions (questions 1 and 33 to 36), vitality (questions 23, 27, 29 and 31), social functioning (questions 20 and 32), mental health (questions 24 to 26, 28 and 30), and one extra question (question 2) not included in the total score. The score for each dimension varies from 0 to 100, with zero being the worst possible health condition and 100 being the best possible health condition^22^. The score was calculated according to the scoring rules of the RAND 36 Health Survey item 1.0, in two phases: 1) all of the items were scored on a scale of 0 to 100; and 2) the mean of the items of each dimension were calculated to create the eight scores of the scale. Any unanswered questions were not included in the calculation. At last, the scores for each dimension represent the mean of all answered items^23^.

### World Health Organization Quality of Life - bref (WHOQOL-bref)

The WHOQOL-bref questionnaire is an abbreviated version of the WHOQOL-100^24^ that has been adapted to Brazilian-Portuguese^25^. It contains 26 questions, including 2 general questions, and the remaining 24 questions representing each of the 24 aspects of the original instrument. It is divided into four domains: physical health (questions 3, 4, 10 and 15 to 18), psychological (questions 5, 6, 7, 11, 19 and 26), social relationships (questions 20 to 22), and environment (questions 8, 9, 12 to 14 and 23 to 25). The WHOQOL-bref scores are calculated according to an algorithm^26^ that considers the number of answered questions in each of the domains and standardizes the scores of all domains from zero to 100, with zero being the worst possible health condition and 100 being the best health condition. The algorithm inverts the score values for questions 3, 4, and 26 to calculate the final score^25^^,^ 27 ^,^ 28.

### Functional Assessment of Cancer Therapy - Breast plus Arm Morbidity (FACT-B+4)

The breast cancer-specific questionnaire FACT-B+4 consists of 36 questions, 27 of which refer to overall QoL and 9 to specific problems of patients with breast cancer^29^. In 2001, a four-question subscale was added to the FACT-B questionnaire to assess arm morbidity in patients submitted to breast surgery^30^. The FACT-B+4 has been adapted into Brazilian-Portuguese^31^. It is divided into six scales with independent scores: physical well-being ranging from 0 to 28 (questions GP1 to GP7), social/family well-being ranging from 0 to 28 (questions GS1 to GS7), emotional well-being ranging from 0 to 24 (questions GE1 to GE6), functional well-being ranging from 0 to 28 (questions GF1 to GF7), breast cancer subscale ranging from 0 to 36 (questions B1 to B9) and arm subscale ranging from 0 to 20 (questions B3 and B10 to B13). The answers are presented on a five-point Likert scale. The score is calculated separately for each scale by adding up the points for each question. The values for some questions (GP1 to GP7, GE1, GE3 to GE6, B1 to B3, B5 to B8, B10 to B13) are inverted in the calculation of the final score. When there were any unanswered questions, the mean of the answered questions was considered for that scale. The results are added to obtain the final total score ranging from 0 to 164. The higher the score is, the better the patient's QoL^29^^,^ 30.

### Global Perceived Effect scale (GPE)

For this research, the GPE scale^32^ was adapted to assess the patient's level of perception of recovery since the day of diagnosis with breast cancer. The guiding question was "Compared to when you received your diagnosis, how would you describe your quality of life these days?". It is an 11-point numerical scale (-5 to 5), with -5 being vastly worse; 0 being no change; and 5 being complete recovery. The higher the score is, the better the recovery from the condition^32^.

### Pain Numerical Rating scale (PNR)

The five-point adapted PNR scale^33^ was used to verify the patient's degree of understanding regarding the QoL questionnaires. The guiding question is: "Did you understand what was asked in the questionnaire?" The minimum value is 0, meaning "I did not understand anything", and the maximum value is 5, meaning "I understood perfectly and did not have any questions"^33^.

### Procedures

The researcher collected the participants' sociodemographic and clinical data and administered the QoL questionnaires at baseline. After that, the participants were informed of the subsequent days when the questionnaires would be administered over the phone, i.e. 48 hours and 30 days after the first session. The 48-hour interval between the first and second session was established to avoid significant changes in the patient's QoL, thus allowing the evaluation of the test-retest reproducibility of the questionnaire. The 30-day interval between the first and third session was established to allow sufficient time for changes in QoL and thus test the responsiveness of the questionnaires^34^.

### Statistical analysis

The assessments of the measurement properties, described in detail in Table 1, were conducted according to procedures recommended by Maher et al.^11^ and Terwee et al.^34^.

**Table 1:** Measurement properties tested.

Measurement properties	
Internal consistency	The homogeneity of the items of the questionnaire was tested using Cronbach’s alpha^11,35^ and Cronbach’s alpha if an item deleted. The Cronbach alpha values are considered adequate when equal to or greater than 0.70 and less than 0.95^11,35^.
Reproducibility	The term reproducibility incorporates two measurement properties: reliability and agreement. Reliability was tested using Type 2,1 Intraclass Correlation Coefficient (ICC_2,1_) with 95% confidence intervals (CIs). An ICC of less than 0.40 represents poor reliability; between 0.40 and 0.75 represents moderate reliability; between 0.75 and 0.90, substantial reliability; and greater than 0.90, excellent reliability. Agreement was measured using the following measurements: Standard Error of the Measurement (SEM)^36^ and Smallest Detectable Change (SDC)^11,35^. The SEM was calculated by the ratio of the standard deviation of the mean difference to the square root of two. The percentage of the SEM related with the total score of the questionnaire can be interpreted as follows: =5%: very good; >5% and =10%: good; >10% and =20%: doubtful and >20%: negative^37^. The SDC was calculated using the formula SDC=1.645 × v2 x SEM, with 90% CI, which reflects the smallest detectable change in an individual’s score. Thus, it can be interpreted that values above the SDC describe a change in the individual’s score above the error of the measurement^35^.
Construct validity	We correlated the domains with the most similarities, e.g. the SF-36 dimensions physical functioning, role-physical, role-emotional, and social functioning with the FACT-B+4 scales functional well-being, physical well-being, emotional well-being, and social/family well-being, respectively, and the WHOQOL-bref domains physical health, psychological, and social relationships with the FACT-B+4 scales physical well-being, emotional well-being, and social/family well-being, using Pearson’s correlation test (r). When r<0.30, the correlation was considered weak, when r=0.30 and <0.60 the correlation was considered moderate and when r=0.60 the correlation was considered good^36^. It is expected that the generic quality of life questionnaires SF-36 and WHOQOL have a positive correlation with the FACT-B+4 with r=0.60, assuming that the construct of the evaluated domains of the three questionnaires were similar.
Responsiveness	The analysis of the responsiveness was based on the participants who showed clinical changes, considering a two-point change (negative or positive) in the GPE scale. The internal responsiveness was assessed by calculating the effect size (ES: mean of difference between initial assessment and 30-day follow-up, divided by the standard deviation of the initial assessment) with 84% CI. We chose 84% CI to allow a direct comparison of the ES of different instruments since CIs that do not exceed 84% are equivalent to Z scores at 95%^38,39^. A value for ES =0.20 represents a change of approximately 1/5 of the standard deviation at the beginning of treatment and it is considered small. A value of 0.50 is considered moderate and a value =0.80 is considered large^40^. The external responsiveness was measured by two tests: 1) Pearson’s Correlation test to determine the correlation between the initial and 30-day assessments of the dimensions of the SF-36^22^, WHOQOL-bref^25^, FACT-B+4^29^ and the GPE scale^32^ assessed on the 30-day assessment session. This type of responsiveness test compares the instruments’ sensitivity to change in relation to a global measurement of quality of life; and 2) the construction of ROC (Receiver-Operator Characteristics) curves using the differences between the initial and 30-day assessments of the SF-36, WHOQOL-bref, FACT-B+4 and the GPE scale dichotomized in patients who changed their quality of life status. The cut-off point to categorize change was based on the number of women who changed their quality of life considering a two-point variation in the GPE scale assessed in the 30-day assessment session. The analysis was based on the area under the curve (AUC) and values of 0.70^35^ or more were considered responsive. This type of responsiveness measures the questionnaire’s ability to distinguish patients who changed quality of life status from those who did not^11,35^.
Floor and ceiling effects	These measurements were calculated by the percentage of patients who achieved the maximum score (ceiling) or the minimum score (floor). These effects are considered when 15% of respondents reach the ceiling or floor scores, leading to implications on the questionnaire’s reproducibility and responsiveness^11,35^.

## Results

A total of 111 eligible women were invited to take part in the study: 5 women declined to answer the questionnaires and 106 women agreed to participate. Of the 106 participants, 99 responded to the second assessment session after 48 hours and 94 responded to the third assessment session after 30 days. These drop outs were caused by side effects of chemotherapy, pneumonia associated with hospital stay, low immunity, infection, necrosis of surgical wound, and second surgery. Table 2 shows the clinical and demographic characteristics, and Table 3 shows the scores for the QoL questionnaires applied in the three assessment sessions. The postoperative period ranged from 3 days to 4 years.

**Table 2:** Characteristics of study participants.

Variables	Baseline (n=106)
**Age** (years), mean (SD)	49.2 (9.6)
**Height **(m), mean (SD)	1.6 (0.1)
**Weight** (Kg), mean (SD)	71.2 (13.3)
**BMI **(kg/m^2^), mean (SD)	27.3 (4.3)
**Marital status**, n (%)	
single	27 (25.5)
married	61 (57.5)
divorced	13 (12.3)
widow	5 (4.7)
**Educational level**, n (%)	
Primary education	12 (11.3)
Secondary education	24 (22.7)
Tertiary education	70 (66)
**P** **ostoperative date **(weeks), mean (SD)	32.7 (50.2)
**Metastasis** ^1^, n (%)	26 (24.5)
**Type of surgery**, n (%)	
Radical Mastectomy	15 (14.2)
Modified Radical Mastectomy	68 (64.2)
Quadrantectomy	23 (21.7)
**Axillary dissection** ^2^, n (%)	94 (88.7)
**Type of ** **axillary dissection** ^1^ n (%)	
Sentinel node	24 (22.6)
Partial axillary dissection	22 (20.8)
Total axillary dissection	47 (44.3)
**Lymphedema**, n (%)	18 (17)
**Fibrous cord**, n (%)	31 (29.2)
**Breast reconstruction** ^1^, n (%)	58 (54.7)
**Type of reconstruction** ^1^, n (%)	
Silicone	34 (32.1)
Tissue expander	23 (21.7)
None	48 (45.3)
**Questionnaire that best represented the ** **QoL** ^1^, n (%)	
SF-36	14 (13.2)
WHOQOL-bref	34 (32.1)
FACT-B+4	57 (53.8)

BMI (body mass index), QoL (quality of life), SF-36 (Medical Outcomes Study 36 - Item Short-Form Health Survey), WHOQOL-bref (World Health Organization Quality of Life - bref), FACT-B+4 (Functional Assessment of Cancer Therapy - Breast plus Arm Morbidity). ^1^Missing data (%): Metastasis (2.8), Type of axillary dissection (0.9), Breast reconstruction (0.9), Type of reconstruction (0.9), Questionnaire that best represented the QoL (0.9); ^2^Patients who did not undergo axillary dissection (11.3%).

**Table 3:** Scores of quality of life questionnaires and scales used in the study in the three assessment sessions, in mean and standard deviation.

Variables	Baseline (n=106)	48 hr after baseline (n=99)	30 days after baseline (n=94)
**SF-36 - Dimensions**			
Physical functioning (0-100)	70.0 (36.2)^1^	70.0 (25)^1^	75.0 (31.2)^1^
Role-physical (0-100)	0.0 (25)^1^	0.0 (0.0)^1^	0.0 (56.2)^1^
Role-emotional (0-100)	66.6 (100)^1^	33.3 (100)^1^	100.0 (66.7)^1^
Bodily pain (0-100)	61.0 (20.7)	62.0 (20.3)	64.8 (18.0)
General health perceptions (0-100)	68.0 (19.2)	70.3 (19.0)	72.1 (18.3)
Vitality (0-100)	65.3 (25.9)	61.4 (23.3)	71.2 (22.7)
Social functioning (0-100)	62.5 (37.5)^1^	62.5 (25.0)^1^	75 (28.1)^1^
Mental health (0-100)	72.0 (28.0)^1^	68.4 (18.0)	69.6 (16.3)
**WHOQOL-bref - Domains**			
Physical health (0-100)	50.6 (17.2)	60.3 (16.4)	63.1 (16.2)
Psychological (0-100)	67.4 (16.8)	70.8 (20.8)^1^	68.6 (15.9)
Social relationships (0-100)	66.7 (19.7)	65.9 (17.9)	66.6 (18.7)^1^
Environment (0-100)	68.9 (12.7)	67.8 (12.8)	67.2 (12.5)^1^
**FACT-B+4 - ** **Scales**			
Physical well-being (0-28)	21.0 (7.2)^1^	21.0 (8.0)^1^	23.0 (7.3)^1^
Social/family well-being (0-28)	22.0 (7.2)^1^	19.8 (8.0)^1^	21.0 (8.0)^1^
Emotional well-being (0-24)	20.0 (6.0)^1^	20.0 (5.0)^1^	20.0 (5.0)^1^
Functional well-being (0-28)	17.8 (5.7)	17.1 (4.9)	18.0 (7.0)^1^
Breast cancer subscale (0-36)	22.3 (5.7)	23.0 (7.0)^1^	25.0 (7.2)^1^
Arm subscale (0-20)	14.1 (4.3)	14.9 (3.9)	15.6 (3.7)
FACT-B+4 total score (0-164)	101.2 (17.6)	100.3 (19.1)	103.5 (19.0)
**GPE** (–5 a +5)	2.6 (2.3)	2.6 (2.4)	2.6 (2.4)
**PNR**			
SF-36 (1-5)	4.5 (0.7)	4.6 (0.6)	4.6 (0.6)
WHOQOL-bref (1-5)	4.6 (0.6)	4.6 (0.5)	4.6 (0.5)
FACT-B+4 (1-5)	4.6 (0.5)	4.7 (0.5)	4.7 (0.5)

SF-36 (Medical Outcomes Study 36 - Item Short - Form Health Survey), WHOQOL-bref* (*World Health Organization Quality of Life - bref), FACT-B+4 (Functional Assessment of Cancer Therapy - Breast plus Arm Morbidity), GPE (Global Perceived Effect scale), PNR (Pain Numerical Rating scale). ^1^Data expressed as median and interquartile range.

Regarding acceptance and preference for the questionnaire that best represented QoL, 53.8% of the participants chose the FACT-B+4 (Table 2). Regarding ease of comprehension of the questionnaires, the means were similar (Table 3).

In the assessment of the internal consistency, Cronbach's alpha for all of the instruments was adequate, with the exception of: the social functioning dimension of the SF-36; the social relationships domain of the WHOQOL-bref, with the highest value of Cronbach's alpha if item deleted reached when question 21 was deleted; and the emotional well-being scale and breast cancer subscale of the FACT-B+4, with no change when using Cronbach's alpha if item deleted (Table 4).

**Table 4:** Values of internal consistency, reproducibility and floor or ceiling effects.

Instruments	Internal consistency	Reproducibility			Floor or ceiling effects	
	Cronbach’s alpha (Cronbach’s alpha if an item was deleted)	ReliabilityICC2,1 (95% CI)	Agreement SEM (%)	Agreement SDC	Floor (%)	Ceiling (%)
**SF-36 - Dimensions**						
Physical functioning (0-100)	0.88 (0.85-0.87)	0.77 (0.68 to 0.84)	11.28 (11.28)	26.24	0.0	6.6
Role-physical (0-100)	0.91 (0.87-0.89)	0.55 (0.40 to 0.68)	23.24 (23.24)	54.06	68.9	13.2
Role-emotional (0-100)	0.88 (0.77-0.91)	0.39 (0.21 to 0.54)	34.52 (34.52)	80.30	33	44.3
Bodilypain (0-100)	0.76 (-)^1^	0.58 (0.42 to 0.70)	15.17 (15.17)	35.28	0.9	5.7
General health perceptions (0-100)	0.70 (0.56-0.69)	0.74 (0.63 to 0.82)	8.82 (8.82)	20.51	0.0	7.5
Vitality (0-100)	0.82 (0.75-0.80)	0.73 (0.62 to 0.81)	10.66 (10.66)	24.81	0.0	1.9
Social functioning (0-100)	0.56 (-)^1^	0.52 (0.37 to 0.66)	16.73 (16.73)	38.91	0.9	17
Mental health (0-100)	0.82 (0.77-0.81)	0.71 (0.60 to 0.78)	10.37 (10.37)	24.12	0.0	0.0
**WHOQOL-bref - ** **Domains**						
Physical health (0-100)	0.83 (0.76-0.84)	0.80 (0.72 to 0.87)	7.39 (7.39)	17.18	0.0	0.9
Psychological (0-100)	0.78 (0.74-0.80)	0.87 (0.81 to 0.91)	6.06 (6.06)	14.10	0.0	0.9
Social relationships (0-100)	0.68 (0.47-0.78)	0.76 (0.66 to 0.82)	9.46 (9.46)	22.01	0.9	5.7
Environment (0-100)	0.75 (0.70-0.75)	0.80 (0.71 to 0.87)	5.77 (5.77)	13.43	0.0	0.0
**FACT-B+4 - ** **Scales**						
Physical well-being (0-28)	0.75 (0.68-0.76)	0.62 (0.50 to 0.73)	2.97 (10.60)	6.93	0.0	3.8
Social/family well-being (0-28)	0.85 (0.80-0.88)	0.76 (0.60 to 0.86)	2.46 (8.78)	5.73	0.0	10.4
Emotional well-being (0-24)	0.67 (0.57-0.67)	0.72 (0.61 to 0.80)	1.79 (7.45)	4.19	0.0	11.3
Functional well-being (0-28)	0.84 (0.80-0.85)	0.62 (0.50 to 0.73)	3.25 (11.60)	7.57	0.9	1.9
Breast cancer subscale(0-36)	0.66 (0.60-0.67)	0.71 (0.60 to 0.80)	2.94 (8.16)	6.87	0.0	0.0
Arm subscale (0-20)	0.84 (0.79-0.85)	0.75 (0.65 to 0.82)	2.02 (10.10)	4.71	0.9	9.4
FACT-B+4 total score (0-164)	0.88 (0.87–0.89)	0.86 (0.80 to 0.90)	7.07 (4.31)	16.48	0.0	0.0

ICC (Intraclass Correlation Coefficient), CI (confidence interval), SEM (standard error of the measurement), SDC (smallest detectable change), SF-36 (Medical Outcomes Study 36 - Item Short - Form Health Survey), WHOQOL-bref (World Health Organization Quality of Life - bref), FACT-B+4 (Functional Assessment of Cancer Therapy - Breast plus Arm Morbidity). ^1^Insufficient number of items for calculating Cronbach's alpha if an item was deleted.

Considering reliability, the SF-36 had six dimensions with moderate reliability, the WHOQOL-bref had substantial reliability in all domains, and the FACT-B+4 had five scales with moderate reliability (Table 4). In most dimensions of the SF-36, agreement was classified between doubtful and negative; the WHOQOL-bref had good agreement in all domains; and the agreement levels of the FACT-B+4 varied from very good to doubtful (Table 4). Regarding the floor or ceiling effects, values above 15% were only found in three dimensions of the SF-36, with floor effect in the role-physical and role-emotional dimensions and ceiling effect in the role-emotional and social functioning dimensions (Table 4).

For the construct validity, the correlations between the scales of the FACT-B+4 and the SF-36 varied from good to moderate (correlation between role-physical dimension of the SF-36 and physical well-being scale of the FACT-B+4: r=0.31, p=0.001; correlation between role-emotional dimension of the SF-36 and emotional well-being scale of FACT-B+4: r=0.41, p=0.000; correlation between physical functioning dimension of the SF-36 and functional well-being scale of FACT-B+4: r=0.39, p=0.000). The association of the scales of the FACT-B+4 and WHOQOL-bref showed good correlation (correlation between physical health domain of the WHOQOL-bref and physical well-being scale of FACT-B+4: r=0.69, p=0.000; correlation between social relationships domain of the WHOQOL-bref and social/family well-being scale of FACT-B+4: r=0.62, p=0.000; correlation between psychological domain of the WHOQOL-bref and emotional well-being scale of FACT-B+4: r=0.61, p=0.000).

In the assessment session after 30 days, 62 patients had changes <2 points and 32 patients had clinical changes ≥2 points in the GPE scale. The analysis of responsiveness considered the data from these 32 patients. Regarding internal responsiveness, the SF-36 showed moderate ES in all dimensions except physical functioning and general health perceptions, which had small ES, and bodily pain, which had large ES. The WHOQOL-bref showed small ES in all domains, except physical health, with moderate ES. The FACT-B+4 showed moderate ES in all scales except social/family well-being, emotional well-being, and functional well-being, which had small ES. With 84% CI, there was no difference between similar domains, i.e. in all comparisons there was overlapping between the CIs. For example, the role-physical dimension of the SF-36 presented ES=0.29 with 84% CI of 0.04 to 0.54 which overlapped the CI of the physical health domain of the WHOQOL-bref, with ES=0.53 and 84% CI of 0.24 to 0.80, and of the physical well-being scale of the FACT-B+4, with ES=0.33 and 84% CI of 0.02 to 0.63.

In the external responsiveness assessment using ROC curve analysis, all dimensions of the SF-36 were responsive, except for physical functioning, role-physical, and role-emotional. In the WHOQOL-bref, all domains had values above 0.70. The physical well-being, functional well-being, and total score scales of the FACT-B+4 were responsive. The Pearson correlation analysis showed a significant and moderate correlation in the dimensions bodily pain, general health perceptions, vitality, and mental health of the SF-36. The WHOQOL-bref showed significant good and moderate correlation for the domains psychological and social relationships, respectively. The FACT-B+4 showed a moderate correlation for the functional well-being and total score scales of the FACT-B+4 (Table 5).

**Table 5:** Internal and external responsiveness.

Instruments	Internal responsiveness	External responsiveness	
	ES (84% CI)(n=32)	AUC¹ (95% CI)(n=32)	r (p)(n=32)
**SF-36 - Dimensions**			
Physical functioning (0-100)	0.11 (–0.23 to 0.45)	0.49 (0.28 to 0.70)	0.17 (0.35)
Role-physical (0-100)	0.29 (0.04 to 0.54)	0.49 (0.27 to 0.71)	–0.04 (0.81)
Role-emotional (0-100)	0.26 (0.01 to 0.52)	0.58 (0.35 to 0.80)	0.08 (0.68)
Bodilypain (0-100)	0.98 (0.68 to 1.27)	0.71 (0.52 to 0.89)	0.37 (0.03)**
General health perceptions (0-100)	–0.26 (–0.52 to –0.01)	0.71 (0.52 to 0.89)	0.42 (0.01)**
Vitality (0-100)	0.39 (0.13 to 0.65)	0.75 (0.58 to 0.92)	0.36 (0.38)**
Social functioning (0-100)	0.41 (0.16 to 0.67)	0.71 (0.50 to 0.92)	0.34 (0.06)
Mental health (0-100)	0.36 (0.14 to 0.58)	0.86 (0.68 to 1.00)	0.53 (0.00)*
**WHOQOL-bref - ** **Domains** ****			
Physical health (0-100)	0.53 (0.24 to 0.80)	0.73 (0.54 to 0.92)	0.27 (0.14)
Psychological (0-100)	0.02 (–0.11 to 0.17)	0.88 (0.76 to 0.99)	0.61 (0.00)*
Social relationships (0-100)	0.12 (–0.06 to 0.30)	0.80 (0.65 to 0.95)	0.44 (0.01)**
Environment (0-100)	0.00 (–0.19 to 0.21)	0.71 (0.52 to 0.89)	0.32 (0.07)
**FACT-B+4 - Scales**			
Physical well-being (0-28)	0.33 (0.02 to 0.63)	0.73 (0.54 to 0.90)	0.33 (0.06)
Social/family well-being (0-28)	–0.11 (–0.28 to a 0.05)	0.60 (0.40 to 0.79)	0.30 (0.90)
Emotional well-being (0-24)	0.17 (–0.08 to 0.44)	0.58 (0.37 to 0.78)	0.22 (0.22)
Functional well-being (0-28)	0.07 (–0.18 to 0.32)	0.86 (0.72 to 0.99)	0.59 (0.00)*
Breast cancer subscale (0-36)	0.37 (0.13 to 0.60)	0.51 (0.28 to 0.74)	–0.03 (0.86)
Arm subscale (0-20)	0.36 (0.11 to 0.60)	0.45 (0.23 to 0.66)	–0.25 (0.17)
FACT-B+4 total score (0-164)	0.22 (–0.01 to 0.47)	0.71 (0.51 to 0.91)	0.40 (0.02)**

SF-36 (Medical Outcomes Study 36 - Item Short - Form Health Survey), WHOQOL-bref (World Health Organization Quality of Life - bref), FACT-B+4 (Functional Assessment of Cancer Therapy - Breast plus Arm Morbidity), ES (Effect size), AUC (area under the curve), CI (Confidence interval). ¹Cutoff for improvement =2 in the Global Perceived Effect scale; *Statistically significant correlations (p<0.01), **Statistically significant correlations (p<0.05).

## Discussion

Most of the domains of the SF-36, WHOQOL-bref, and FACT-B+4 showed acceptable values for the measurement properties. All instruments showed good comprehension represented by similar means. With regard to the questionnaire which best-represented QoL, 53.8% of the participants chose the FACT-B+4, possibly due to the fact that this instrument included specific questions to breast cancer and upper limb limitations.

In our study, the SF-36 showed adequate Cronbach's alpha in all dimensions except social functioning. Similar studies with different samples were found in the literature. In a population of Chinese medical students, Cronbach's alpha ranged from 0.63 to 0.82, with the lowest value in the social functioning dimension. This result may be due to the fact that the items of this dimension are not sensitive to cultural variations and may need to be adapted to the characteristics of the target population^41^. In Chinese patients with chronic diseases, Cronbach's alpha ranged from 0.54 to 0.93, with the lowest values in the dimensions bodily pain (0.54) and social functioning (0.62)^42^. In contrast, in a study with a population of 50 healthy individuals and 80 patients with chronic disease, Cronbach's alpha ranged from 0.72 to 0.89^43^.

Moderate reliability was found in all dimensions of the SF-36 except role-emotional, which had poor reliability, making it impossible to obtain similar results among the participants of this study. Other studies found in the literature show substantial to excellent reliability. In a population of Chinese patients with chronic disease, ICC values ranged from 0.83 to 0.96^42^. In a sample of 130 Arabic individuals, ICC ranged from 0.95 to 0.98^43^. However, both of these studies may have overestimated the results because they did not report the type of ICC used. That may be the reason why these studies found higher ICC values than those in our study. For agreement, the present study found high standard error of measurement (SEM) values (most of the dimensions showed values >10% and ≤20%) and smallest detectable change (SDC) ranging from 20.51 to 80.30, characterizing the SF-36 as having doubtful agreement.

We found the presence of floor effect in the dimensions role-physical and role-emotional and the presence of ceiling effect in the dimensions role-emotional and social functioning. These specific dimensions were probably unable to detect change in the patients' health condition, with implications on reproducibility and responsiveness. For construct validity, analyzed by the combination of dimensions from the SF-36 and the FACT-B+4, the results indicated a significant correlation in all dimensions except the social functioning dimension of the SF-36. No studies were found that conducted a similar correlation between these two questionnaires.

The assessment of the internal responsiveness showed that responsiveness ranged from small to large. Considering external responsiveness, the SF-36 was characterized as a responsive instrument. Furthermore, a significant correlation was found between the dimensions that had AUC values above 0.70. The SF-36 showed at least one dimension with inadequate values in all measurement properties tested. This result implies that the SF-36 should not be used to evaluate QoL in patients with breast cancer.

The WHOQOL-bref presented adequate internal consistency in most of the domains, except for the social relationships domain. No studies were found on assessment of the measurement properties of the WHOQOL-bref in patients with breast cancer. In other populations, studies that tested the internal consistency of the WHOQOL-bref found similar values^25^^,^ 28 ^,^ 44 ^-^ 46. One study in which the internal consistency of the WHOQOL-bref was compared to that of the WHOQOL-100 found a higher Cronbach's alpha. Thus, the low value of the abbreviated questionnaire can be explained by the low number of questions in the social relationships domain given that Cronbach's alpha is dependent on the number of items of a scale^25^^,^ 34.

Reliability was substantial in all domains of the WHOQOL-bref. These results are similar to those of one study^28^, in which the values varied from substantial to excellent. However, this study^28^ does not report the type of ICC used. For the agreement, good SEM values were found and an SDC of 13.43 to 22.01, characterizing the WHOQOL-bref as having good agreement.

There were no floor or ceiling effects. The construct validity presented a good correlation. No study was found that conducted a similar correlation between the two questionnaires. Internal responsiveness showed small responsiveness in most of the domains. A study with smokers also found small responsiveness for all domains except the psychological domain^44^. The assessment of the external responsiveness by the AUC showed responsiveness in all domains. However, only the psychological and social relationship domains showed significant correlation. After the analysis, the WHOQOL-bref can be used to assess QoL in patients with breast cancer given that the measurement properties were adequate and the instrument was able to detect clinical changes over time.

The FACT-B+4 showed adequate values for internal consistency, with the exception of the emotional well-being scale and the breast cancer subscale. Other studies found lower internal consistency values for the same scales, suggesting that there is no homogeneity in these scales. For example, in the original validation study of the arm subscale of the FACT-B+4, the internal consistency ranged from 0.62 to 0.83^30^; in a sample of breast cancer patients before surgery with upper limb lymphedema, the internal consistency varied from 0.52 to 0.92^47^.

For reliability, most scales showed moderate reliability. Conflicting results were found in a sample of patients with lymphedema, with reliability ranging from 0.40 to 0.88^47^, and the study did not report the type of ICC used. The agreement values for the scales of the FACT-B+4 were characterized between good and doubtful. For the FACT-B+4 total score, a very good agreement was observed. Floor or ceiling effects were not observed. In contrast, another study on women with breast cancer showed ceiling effects in the physical well-being and social/family well-being scales and the arm subscale^47^. For construct validity, the FACT-B+4 presented better correlation with the WHOQOL-bref, with good correlation between all scales.

The assessment of internal responsiveness showed small to moderate responsiveness. External responsiveness, based on the analysis of the AUC, was only found for the physical well-being, functional well-being, and total score scales. The correlation analysis showed moderate correlation for the functional well-being scale and total score.

The measurement of QoL is important to understand how functional impairment interferes in the daily activities of women undergoing treatment for breast cancer. Considering that the assessment of QoL is multidimensional^48^^,^ 49, with different meanings depending on the variety of life contexts, maintenance of functional capacity, general satisfaction, personal fulfillment, and social interaction^48^^,^ 49, physical therapists should investigate QoL with the goal of improving the treatment and monitoring the evolution of the clinical condition, which contributes to prevention interventions or treatment directions^6^^,^ 50.

Some limitations can be suggested in this study. The inclusion criteria included the largest possible number of women with breast cancer regardless of their phase of treatment. The wide variety in the type of surgery and time since surgery may have become a limitation because a more homogeneous sample in regard to treatment phase or surgery type could have resulted in similar changes in QoL. However, the current sample was based on previous studies^51^^,^ 52. Another limitation was the 30-day interval for the responsiveness assessment. Perhaps if this follow-up time had been longer, greater clinical changes could have occurred and better results could have been found.

Most of the measurement properties tested for the WHOQOL-bref and FACT-B+4 were adequate as was their ability to assess QoL in women with breast cancer. The domains of WHOQOL-bref and FACT-B+4 are interconnected in the measurement of QoL in the studied population. The SF-36 showed inadequacy in agreement and floor and ceiling effects and should not be used to assess QoL in women with breast cancer.
